# Optimization of Optical Properties of Polycarbonate Film with Thiol Gold-Nanoparticles

**DOI:** 10.3390/ma2031193

**Published:** 2009-09-02

**Authors:** Claudio Larosa, Enrico Stura, Roberto Eggenhöffner, Claudio Nicolini

**Affiliations:** 1 Nanoworld Institute – CIRSDNNOB and Biophysics Division, University of Genova, Corso Europa 30, 16132 Genoa, Italy; E-Mails: clarosa@nwi.unige.it (C.L.); estura@nwi.unige.it (E.S.); reggenhoffner@nwi.unige.it (R.E.); 2 Fondazione El.B.A., Piazza SS Apostoli 66, 00100 Roma, Italy

**Keywords:** thiol gold-nanoparticles, polycarbonate, UV-vis plasmon absorption band

## Abstract

A new nanostructured composite film based on thiol gold nanoparticles dispersed in polycarbonate and prepared by evaporating a solution of 1-dodecanthiol gold nanoparticles and polycarbonate was developed for applications as optical lenses. Lenses with superior mechanical properties, coloring and UV ray absorption and with the same transparency as the matrix were obtained. The supporting highly transparent polycarbonate matrix and the chloroform solution of thiol gold nanoparticles, 3 nm mean size, was mixed according to a doping protocol employing different concentrations of thiol gold nanoparticles vs. polycarbonate. The presence of nanoparticles in the polymer films was confirmed by the spectrophotometric detection of the characteristic absorbance marker peak at 540–580 nm. The nanostructured films obtained show a better coverage in the UV-vis range (250–450 nm) even at very low doping ratios, of the order of 1:1,000. These results offer a very promising approach towards the development of efficient nanostructured materials for applications to optical lenses.

## 1. Introduction

Noble metal-nanoparticles are widely studied due to their novel material properties [1]. In particular, gold nanoparticles have chemical and physical properties that may be employed in optical devices [2,3,4] and electronic, catalysis and biosensor technology [5,6,7,8,9]. Dispersions of gold-nanoparticles have found a broad range of applications in bioassays, microscopy and material science. The control of their morphology at the nanoscale is currently one of the most active research objectives. In particular, the optoelectronic and physicochemical properties of nanomaterials are strongly affected by their shape and size, which is derived from a reduction of the number of free electrons in nanoparticles smaller that 5 nm [10]; thus, the particle size distribution and stability must be carefully controlled. Accordingly to these requirements, our choice of gold as starting colloid material was motivated by its chemical and thermal stability and high affinity towards thiol derivatives. Alkanethiols are effective capping reagents to stabilize the small size of gold nanoparticles [11] and to prevent them from aggregation. The incorporation of nanoparticles in a polymer matrix is a field of particular interest for material engineering and for the study of nanoparticle vs. matrix interactions. Various techniques have been developed in recent years to produce a fine dispersion of metals in organic matrices [12,13,14,15,16] in view of their interesting optical, electrical, thermodynamic, catalytic and magnetic properties.

The aim of this work is to investigate optical properties of thiol gold-nanoparticles in polycarbonate pellets, and to evaluate their potential application to lenses and material coloring. Polycarbonate (PC) is an important thermoplastic with remarkable mechanical properties such as miscibility, process ability, water resistance and transparence, due to it is stable amorphous state. Therefore, polycarbonates are largely used in lenses for glasses and were selected as matrix in present work, *i.e.*, as the major component of the nanocomposite. Thus, the optimization of a new method to introduce thiol gold-nanoparticles (TGNPs) at very low concentrations in polycarbonate polymers was investigated by UV-vis absorption, CCD camera and AFM. In this paper, colloidal nanoparticles of gold thiol were prepared by chemical reduction with borohydride, as confirmed by the above techniques and by Rayleigh scattering. It was shown that all nanocomposite samples present characteristic absorption near 530 nm and a better coverage in the ultraviolet range. The coverage offered by a thin film of polycarbonate with very dilute TGNPs (of the order of 0.1‰) is 50% higher than in pure PC in the UV range, a very interesting results for optical device and aerospace plastic components.

## 2. Materials and Methods

### 2.1. Reagents

Hydrogen tetrachloroaurate trihydrate was supplied by Aldrich. Tetraoctylammonium bromide, 1-dodecanethiol, sodium borohydrate, ethanol 98% (v/v) and toluene were all obtained from Sigma. Polycarbonate (Lexan 121 R), 32kDa M_w_, 5 mm^3^ size pellets were supplied by ϑquiplilon, Japan. Chloroform (Sigma Aldrich) was used as solvent for polycarbonate doping assay and in TGNP treatments.

### 2.2. Rayleigh spectroscopy

Dynamic light scattering (DLS) data were acquired with a research-grade goniometer and laser light scattering system (Brookhaven Istrument, BI-200SM), equipped with an argon ion laser operating at the 5145 Å green line. To prepare nanocomposites for Rayleigh scattering, 1 mg of TGNPs was dissolved in 1 mL of chloroform and sonicated for 5 min to disrupt the possible coalescence of TGNP clusters. After filtration with a 0.22 μm Millipore filter paper under pressure, the transparent solution was diluted to 20 mL and the analysis was done at a refractive index of 0.47.

### 2.3. Atomic Force Microscopy (AFM)

A customized atomic force microscopy (SPMagic by Elbatech srl, Italy) was employed to characterize the morphology of TGNPs and of TGNPs/PC films as well as, pure PC for reference. For the preparation of the sample, 1 mg of TGNPs was dissolved in chloroform and diluted to the ratio 1:65,000 (w/v); the sample has been sonicated for 10 min and deposited on a mica surface for solution casting. Eventually, it was evaporated at room temperature. The films of nanocomposite and pure PC films were cut for optical and AFM investigation with a microtome. Finally, the AFM measurements were carried out in tapping mode in air at room temperature by using a NSC18-AIBS tip for TGNPs on mica surface and a NSC15 tip for TGNPs/PC films. The images of the samples were elaborated with freeware VSxM 4.0 software [17].

### 2.4. Spectrophotometric analysis

TGNPs were characterized by spectrophotometric analysis with Jasco model 7800 using a quartz cell with a path length of 1 cm. A solution composed by TGNPs and chloroform with a concentration of 10 μg/mL was used for this analysis. Chloroform background spectra were subtracted by simple UV-vis spectra. To avoid clusters formation, samples were submitted to sonication for 10 min using PBI International model sonic 300 V/T.

## 3. Experimental Section

### 3.1. Preparation of composites

**Figure 1:**
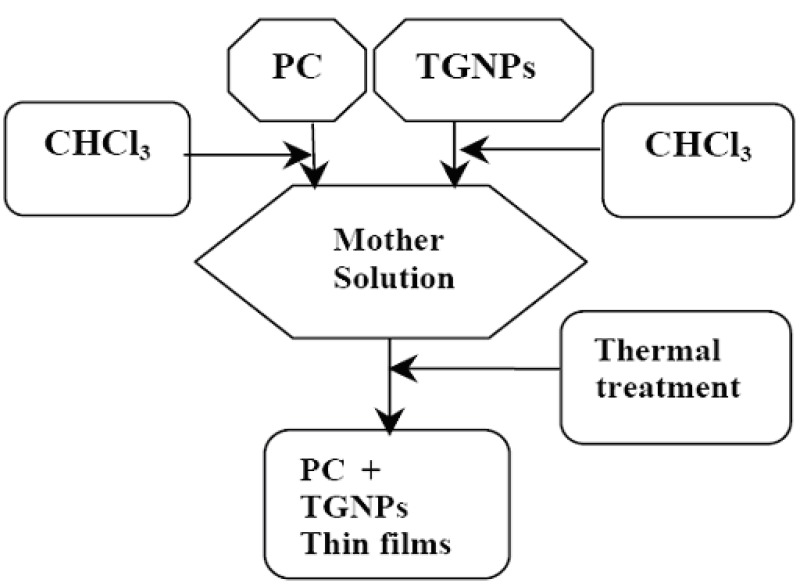
Flow chart of the preparation of polycarbonate nanocomposite. Polycarbonate pellets (PC) and 1-dodecanthiol gold nanoparticles (TGNPs) are dissolved separately in chloroform (CHCl_3_). The two solutions are mixed together to give the solution from which thin films are prepared under the thermal treatment as discussed in the text.

The main stages the composite preparation are outlined in Figure 1. The synthesis of TGNPs was performed as described by Brust *et al.* [18,19] In brief, the method consists of growing the gold clusters simultaneously to self assembled thiol monolayers on surface nuclei. The organic and inorganic phases were mixed and stirred until gold chloride was completely transferred into the organic phase by the surfactant. The organic phase was separated after gold redox and the thiol compounds were added to obtain an Au:thiol molar ratio = 2:1. A fresh aqueous solution of NaBH_4_ was slowly added to the organic phase and stirred for 3 h. After separation and toluene evaporation, we have dispersed TGNPs in ethanol and recovered the final black precipitate by filtering under vacuum with 0.8 μm cellulose acetate filter and stored in dry box. We reduced all the amounts in Ref. [18] by a factor of two; our yield was around 90%. Polycarbonate pellets (PC in Figure 1) were first cleaned with a neutral detergent, followed by rising with distilled water and drying at 120 °C in air circulating oven for 12 h, to remove absorbed water and then dissolved in chloroform.

For the preparation of the solution, 1 mg of nanoparticles was dissolved into 1 mL of chloroform, sonicated for 10 min for disrupting the *islands* and filtered with a 20 µm membrane of porosity. Dissolved TGNPs were added to 10% (w/v) PC/chloroform solution in order to obtain concentrations in the range 0.1 – 10‰. Chloroform, with respect to other disposable solvents, allows one to achieve an intimate contact during the blending procedure. Chloroform was removed by rotavapor at bath temperature of 50 °C; the concentrated solution was placed in a Petri dish (diameter of 80 mm) and dried in an oven at 140 °C for 8 hours. A few samples of the solution and of the dried samples were analyzed by UV-vis spectrometry. The nanocomposite film obtained with above ratio of TGNPs/PC is flexible, transparent, translucent and pink colored under visual inspection; at low TGNPs content, of the order of 1:65,000, TGNPs/PC samples show identical behavior with respect to PC.

### 3.2. UV-vis measurement

Final thin films and samples obtained at intermediate stages in the preparation procedure were analyzed through UV-Vis spectrometry in the range of 300–750 nm. Solutions of TGNPs in chloroform were prepared dissolving 1 mg of TGNPs in 1 mL of chloroform and solutions of TGNPs/PC are obtained by mixing the above solutions with PC dissolved in chloroform. The baseline absorption of pure solvent was also collected. TGNP*s*/PC were previously solved in chloroform and filtered with a 0.22 μm porous membrane; then UV-vis spectrophotometric assay was performed. The above procedures were performed at 24 °C to prevent a too fast chloroform evaporation and vapor densification on the quartz cuvette.

## 4. Results

### 4.1. Rayleigh scattering

**Figure 2:**
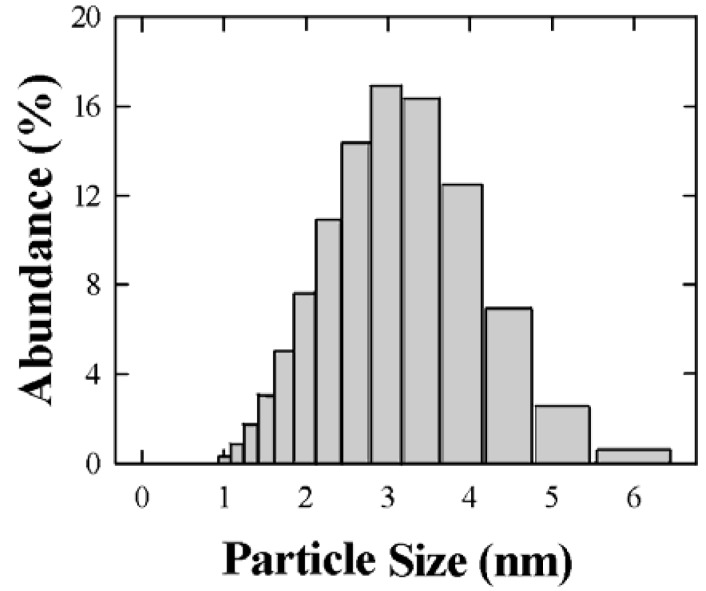
Distribution of nanoparticle sizes obtained by Rayleigh scattering.

In Figure 2, the size distribution of filtered nanoparticles of TGNPs ranges from 1 to 6 nm with an average size around 3.0 nm and a standard deviation of 2 nm. Nanoparticles with this distribution are used for the blending with polycarbonate. Many other syntheses were performed by changing both ratio and reaction time; obtaining reproducible sizes when selecting molar ratio of Au/S = 2:1 and reaction time of 3 h [11,20,21]. Larger sizes, up to 200 nm, are obtained at longer reaction times.

### 4.2. Characterization of nanoparticles

**Figure 3:**
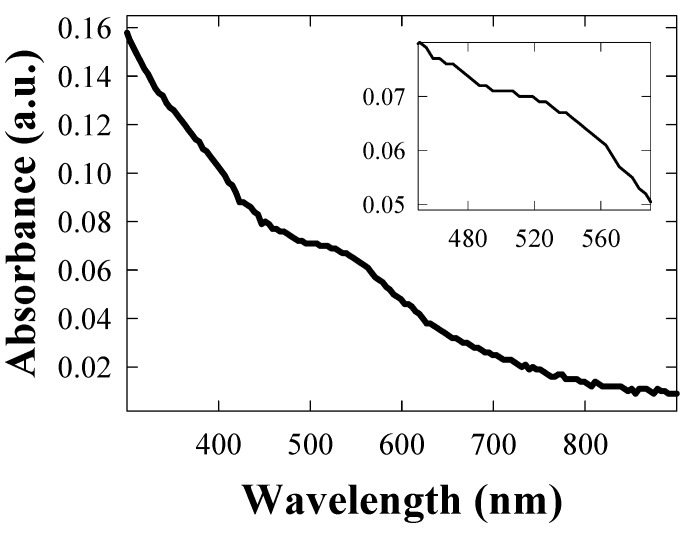
Absorption spectra of TGNPs nanoparticles in chloroform. In the inset, the gold plasmon absorption peak band is reported.

**Figure 4:**
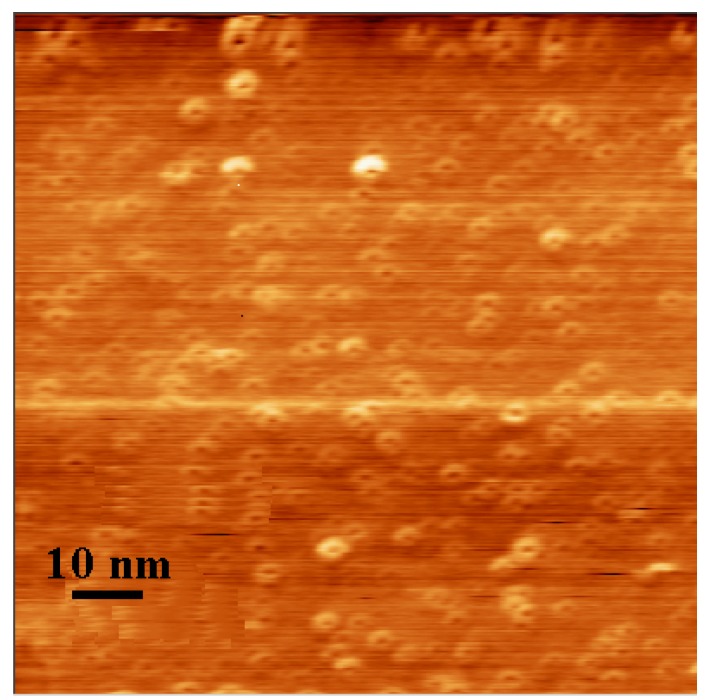
AFM image of TGNPs in chloroform.

The UV-vis absorption spectra of the fresh TGNPs, synthesized as discussed in Section 3.1 above with the molar ratio of Au:S = 2:1 is shown in Figure 3. TGNPs dissolved in chloroform show an absorption band with a peak maximum around 530 nm, superimposed on a decreasing behavior at increasing wavelength. We point out that the fingerprint of TGNPs is still observable at the dilution of 200 ppm by volume. AFM investigation of nanoparticles deposited on a mica surface from a chloroform solution is reported in Figure 4; the geometrical shape of the TGNPs is approximately spherical, with a very low degree of aggregation. Only a few samples with the specific preparation parameter reported above give rise to depositions as regular as reported in Figure 4.

### 4.3. Characterization of polycarbonate film

We prepared films of TGNPs dispersed in polycarbonate with thickness around 15 μm as measured by a CCD camera interfaced with a personal computer running Axio Vision 3.0 software (Carl Zeiss). An active area of 1,152 × 1,242 pixels was examined with 10X optical magnification. With the same apparatus we explored the morphology of TGNPs/PC, that shows traces of film roughness along with a few black spots of particles 3–10 μm in size, irregularly aggregated with elliptic shape and randomly oriented.

**Figure 5:**
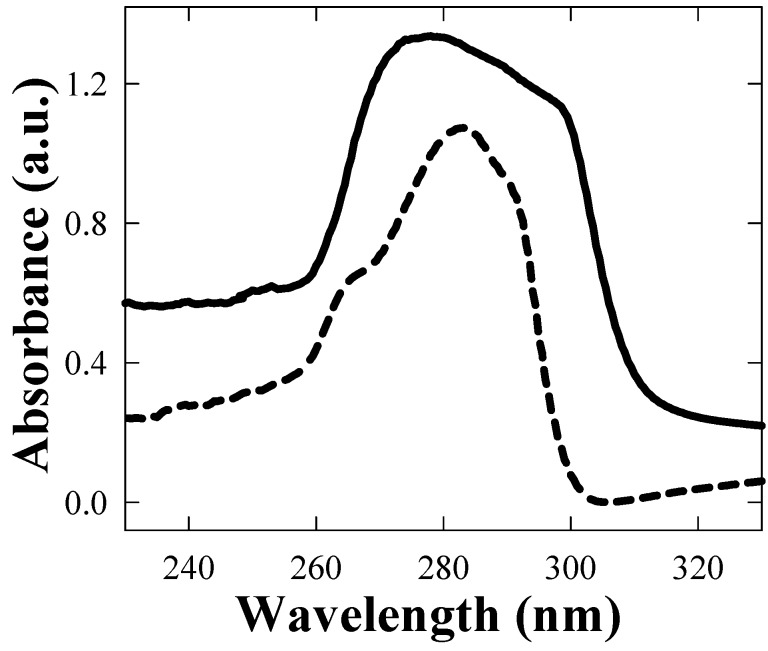
UV-vis spectra at low wave lengths of pure PC ( - - - curve) and of TGNPs/PC dissolved in chloroform (   **———**   curve, 1 ‰).

The spectrum of PC adopted as matrix dissolved in chloroform reported in Figure 5 (dashed curve) shows a narrow absorption band in the 250–280 nm range whereas, in the visible region, absorption intensity was of the order of the detection limit. The full line spectrum is obtained from a solution of TGNPs/PC with 0.1‰ concentration. The spectrum is still dominated by the PC absorbance in the UV range of the Figure 5, but a better absorption coverage, of the order of 20%, is clearly shown by the comparison of the two curves, even at this high dilution of nanoparticles.

**Figure 6:**
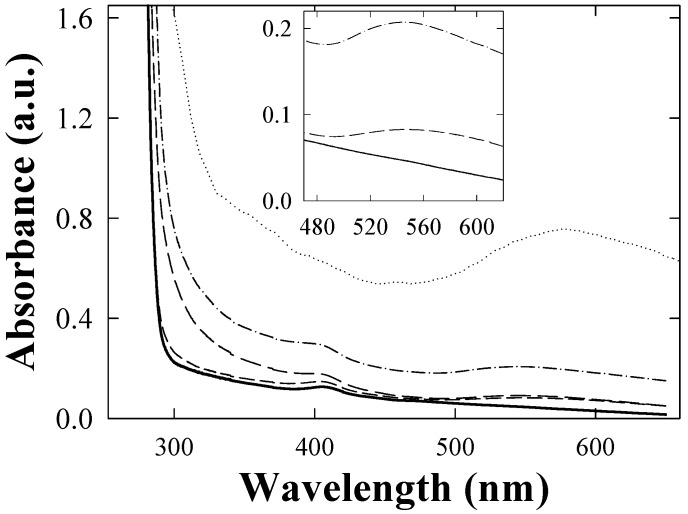
Absorption spectra of TGNPs/PC at growing TGNPs concentration: 0.1‰ solid curve, - - -: 0.5 ‰,  —  ·  —: 1 ‰,  − · −  · −: 2 ‰ and  ············: 5 ‰.

In Figure 6, the absorption spectrum of our polycarbonate films with dispersed TGNPs is reported. As comparisons with Figure 3 and Figure 5 suggest, the low wavelength range spectrum is mostly dominated by the polycarbonate absorption; the fingerprint of the TGNPs is present above 450 nm, approximately. At growing nanoparticle concentration with respect to PC from 1‰ up to 6‰ we observe the increase of TGNP absorption band centered around 545 nm, irrespective of TGNP concentration.

We observe also a absorption peak at 410 nm approximately superimposed on the decreasing trend of the PC wide band. This peak is present even at very low concentration of TGNPs vs. PC and its intensity does not appear to depend critically on this concentration.

**Figure 7:**
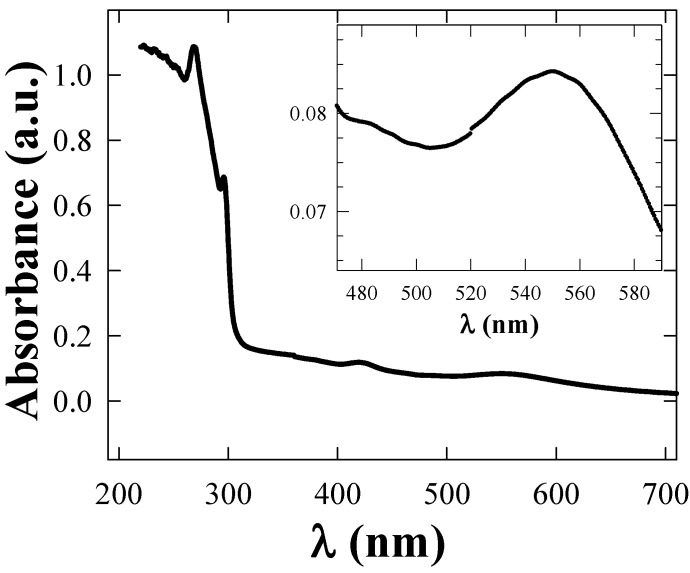
Difference absorption spectrum obtained from a thin film of TGNPs/PC and a PC film used as reference, to eliminate the PC film contribution.

The absorption spectrum of a film of TGNPs/PC at the relative concentration of 2‰ with thickness 10 µm was measured by using a film of pure PC of comparable thickness in the reference holder of the spectrophotometer. Thus, as shown in Figure 7, the absorption can be related only to the contribution of nanoparticles and to their interactions with polycarbonate. The contribution of the TGNPs is appreciable only in the visible range, negligible in the UV where the excess absorption is achieved.

## 5. Discussion

The size of the TGNP distribution obtained by light scattering technique, centered around 3 nm, as reported in Figure 1, is in agreement with the data obtained by AFM measurement reported in Figure 4. In the AFM image, a significant fraction of gold nanoparticles deposited on a mica surface from a chloroform solution appears spherical and with average dimensions compatible with the light scattering observation. A minor fraction of these particles deviates slightly from spherical shape as expected by the difficulties in stabilizing nanoparticles in solution and to completely avoiding aggregation phenomena, in spite that alkanethiol coverage is the most effective agent in favoring uniform nanoparticle dispersions. These residual aggregations are mainly favored by the interchain van der Waals attractive force between alkanethiol chains. The mean diameter of gold nanoparticles achieved by our treatment is approximately equal to 3 nm, *i.e.*, compatible with recent literature values for corresponding TG nanoparticles [18,20]. The UV-vis absorption spectrum of TG nanoparticles in Figure 3 shows a typical broad peak centered at wavelength ranging from 530 nm when dissolved in chloroform and a peak around 545–580 nm when dispersed in PC, as reported in Figure 6. The intensity of the broad band absorption peak superimposed to the decreasing behavior grows at increasing TGNP concentration. Both the decreasing behavior and the broad peak at 545–580 nm is typical of the intense plasmon absorption band of gold nanoparticles [20]. The lower intensities with respect to literature spectra are due to our higher dilution of the TGNPs with respect to both chloroform and PC. From the UV-vis spectra we prove that the introduction of these nanoparticles in polymer, to obtain a nanocomposite material, changes the absorbance profile, increases the absorption in the UV region and is responsible of the absorption in the visible. Since at different doping levels the characteristic absorption remains in the range 530–580 nm, we suggest that the peak is an important marker of the material blending process. The absorption wavelength of the plasmon resonance of nanoparticles depends on the thiol coverage degree and their dispersion in the polymer matrix [23]. The wavelength shift we observe between 530 nm plasmon band resonance of TGNPs in chloroform up to 580 nm of the same resonance in the TGNPs-PC is related therefore to the confinement of the thiol gold particles in PC affecting the interactions with electromagnetic radiation [22,24]. Actually we observe that peak wavelength increases with TGNPs vs. PC concentration up to a 5‰ and, surprisingly decreases at higher concentrations. The absorption peak disappears for dilutions of TGNPs/PC above 1:6,000. The relevance for optical properties of the interactions of PC chains with TGNPs are demonstrated by the differential absorption of TGNPs/PC vs pure PC in the UV wavelength range, shown in Figure 6 for the chloroform solution and, with more evidence, in Figure 7 for the thin films. We observe that the solution prepared after TGNP dissolution in chloroform presents a pink-violet colour estimated from an Ostwald chromatic circle. This colour changes to a uniform pink in the nanocomposite thin film with ratio from 1:100 up to 1:5,000 obtained with a blending method by solvent removal. This suggests the possibility to use this method to colour plastic materials. Opacity in thick films as well as pellets can arise by different mechanisms such as rearrangement of PC chains during solvent evaporation or thermodynamical stabilization of an opaque amorphous or partially crystalline state. Our films appear white and opaque when their thickness exceeds 30 μm.

**Figure 8:**
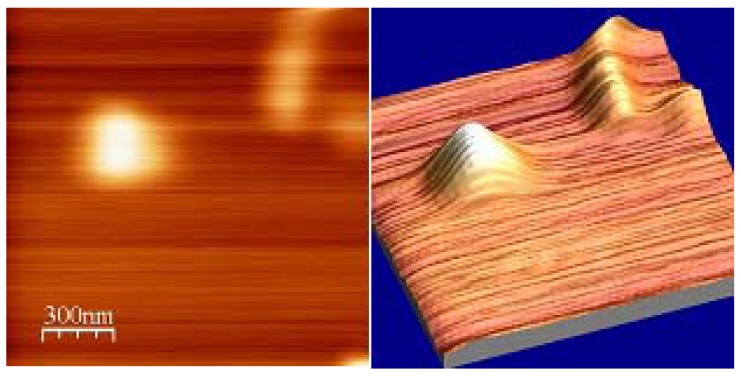
Two and three dimensional AFM images of TGNPs in PC. Scale of 300 nm is common to both figures.

The AFM image of TGNPs/PC thin films reported in Figure 8 shows two different behaviors in the upper with respect to the lower region. The latter is statistically representative of more than 90% of the entire surface of the film, as flat as pure PC thin film surface. The former is a very rare observation of surface corrugation (one wide peak and one slip line was detected over twenty areas of 1 μm^2^ each that were explored). The occurrence of a large spherical TGNPs agglomerate with diameter of 200 nm and a slip line as long as 500 nm is shown in the upper region of the image. The AFM profile analysis indicates that these structures stand up to 5 nm at maximum above the flat surface. It is well known that thermal treatment above 100 °C favors the nanoparticles penetration insides the film and the aggregation of nanoparticles at surface. Our blending films preparation allows a uniform dispersion of nanometric gold particles within the film, however the thermal treatment at 135 °C we used to removed completely chloroform solvent as produced the surface islands shown by AFM images in Figure 8. Further improvements are necessary to avoid thermal treatments in order to obtain corrugation free surfaces for specific applications.

## 6. Conclusions

This paper arose from the need to exploit the unique properties of nanoparticles, specifically in polymer composites as required by factories of polymer materials. Optical properties of well dispersed nanocomposites at very low doping contents showed improved coverage in UV-vis spectra. The nanocomposite film absorbs near UV radiation and, to lower extent, also in the visible range. The advantage of using a solvent to disperse thiol gold-nanoparticles into a polymer for doping is exploited in the present work; in particular we have selected to use polycarbonate since it is widely used for the construction of lenses. Another interesting aspect of this method consists in the nanoparticles/polymer ratio control as we have reported. Moreover, it introduces a simple blending approach to the optimal formation of TGNPs/PC nanocomposite films.
